# Supplementary material for: How strong is strong? The challenge of interpreting network edge weights
Source: PLoS One. 2024 Oct 3;19(10):e0311614. doi: 10.1371/journal.pone.0311614 (PMC11449300; doi:10.1371/journal.pone.0311614)
Supplement: Attachment — Submitted filename: Letter2.pdf [file pone.0311614.s001.pdf]

Summary of revisions to:
How strong is strong? The challenge of interpreting network edge weights

Dear Dr. Cherifi and anonymous reviewer,

Thank you for your very helpful and constructive comments, and for the opportunity to revise and resubmit this manuscript. I have revised the comment to address your concerns, and as a result, believe the new version is significantly improved. Below I summarize my replies and revisions.

1 Editor

No comments

2 Reviewer #1

2.1 I recommend adding a figure that briefly describes the data to demonstrate that it covers a wide range of networks. For instance, you can plot the networks with density on the x-axis, clustering coefficient on the y-axis, each network category (transportation, social, biological, etc.) represented by different shapes (square, circle, triangle, etc.), and the point size representing the number of nodes.

Thanks for this great suggestion. I have added a figure that summarizes these networks' context, size, density, and clustering coefficient. For additional information on the empirical networks included in this corpus, I direct readers to Neal (2024), which is the source of this corpus.

2.2 The analysis is conducted using different statistical significance levels. However, the methods employed involve multiple significance testing, which necessitates corrections such as Bonferroni or False Discovery Rate (FDR). The author should consider performing the analysis with and without multiple testing corrections to observe their effect on Cohen's d and the percentage of ambiguity.

This is a great suggestion! I have expanded the sensitivity tests reported in Figure 5 to include controlling the False Discovery Rate (FDR) at 0.05. As in the other sensitivity tests, all findings replicate, suggesting that the findings are also robust to controlling for type-I error inflation introduced by multiple independent hypothesis tests.

2.3 Line 100: "These two examples point to the ambiguity of information conveyed by edge weights in a weighted network." should be "This example points...".

I have retained the original text here. In this case, the 'two examples' refer to the high-volume ATL-LAX example and the low-volume PDX-PDT example. A parallel construction occurs in the last paragraph before the methods section where 'two examples' refers to the high-sponsorship Merkley-Klobuchar example and the low-sponsorship McConnell-Corker example.

2.4 Line 134: "The formal mathematical specification of DF is provided by" should be "The formal mathematical specification of SDSM is provided by"

Corrected.

References

Neal, Z. P. (2024). The not-so-forbidden triad: Evaluating the assumptions of the strength of weak ties. *Network Science*.
